# The hypothalamic NPVF circuit modulates ventral raphe activity during nociception

**DOI:** 10.1038/srep41528

**Published:** 2017-01-31

**Authors:** Romain Madelaine, Matthew Lovett-Barron, Caroline Halluin, Aaron S. Andalman, Jin Liang, Gemini M. Skariah, Louis C. Leung, Vanessa M. Burns, Philippe Mourrain

**Affiliations:** 1 Department of Psychiatry and Behavioral Sciences, Stanford University, Stanford, CA 94305, USA; 2 Department of Bioengineering and CNC program, Stanford University, Stanford, CA 94305, USA; 3 Department of Chemical and Systems Biology, Stanford University, Stanford, CA 94305, USA; 4 INSERM U1024, Ecole Normale Supérieure Paris, 75005, France

## Abstract

RFamide neuropeptide VF (NPVF) is expressed by neurons in the hypothalamus and has been implicated in nociception, but the circuit mechanisms remain unexplored. Here, we studied the structural and functional connections from NPVF neurons to downstream targets in the context of nociception, using novel transgenic lines, optogenetics, and calcium imaging in behaving larval zebrafish. We found a specific projection from NPVF neurons to serotonergic neurons in the ventral raphe nucleus (vRN). We showed NPVF neurons and vRN are suppressed and excited by noxious stimuli, respectively. We combined optogenetics with calcium imaging and pharmacology to demonstrate that stimulation of NPVF cells suppresses neuronal activity in vRN. During noxious stimuli, serotonergic neurons activation was due to a suppression of an inhibitory NPVF-ventral raphe peptidergic projection. This study reveals a novel NPVF-vRN functional circuit modulated by noxious stimuli in vertebrates.

Neuropeptides are a diverse family of neurotransmitters that influence a wide variety of functions in many species^1^. Despite their importance, many neuropeptides remain poorly characterized, particularly with respect to their behavioral function in vertebrates. Among these are the RFamide peptides, RFRP-1/NPSV and RFRP-3/NPVF, cleaved from a preproprotein encoded by the *npvf* gene. PreproNPVF-derived peptides are produced by neurons in the hypothalamus, and have been analyzed using systemic pharmacology, revealing a role in nociception^2^,^3^. NPVF-derived peptides act through G-protein-coupled receptors and have anti-opioid effects, indicating the pro-nociceptive activity of these neuropeptides. However, the brain regions modulated by NPVF-derived peptides and the neural circuit mechanisms mediating these nociceptive effects are unknown.

The deep location and molecular heterogeneity of hypothalamic neurons have presented challenges for analyzing such cell types in behaving mammals. We took advantage of the fact that neuropeptides and other neuromodulators are well conserved across vertebrates^4^,^5^,^6^, allowing us to analyze these circuits in the brain of larval zebrafish: a small genetically amenable vertebrate with access to deep-brain neurons for optical interrogation^7^. Here, we analyzed the structure of a pain-sensitive NPVF circuit in zebrafish, demonstrating an anatomical and functional link from NPVF neurons to serotonergic neurons in the raphe nucleus. We demonstrated a causative effect between the stimulation of NPVF neurons and the inhibition of ventral raphe neurons, providing evidence for a novel neural circuit involved in pain response in vertebrates.

## Results and Discussion

**Figure 1:**
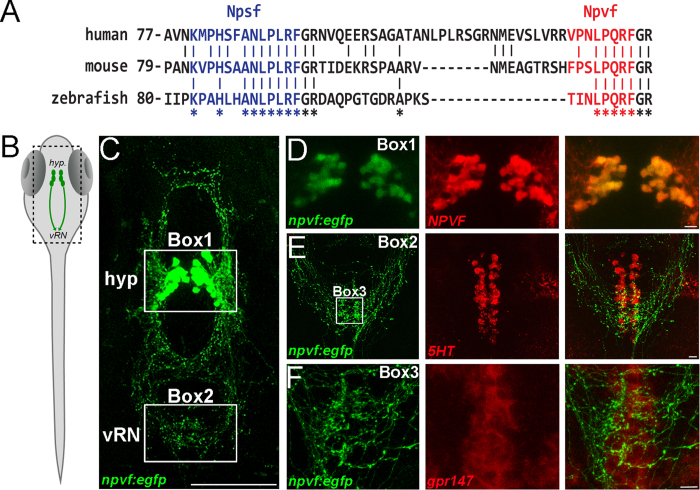
NPVF circuit interacts with the raphe nucleus. (**A**) Alignment of human, mouse and zebrafish NPVF precursors. Multiple sequence alignments show strong conservation in the sequence of putative NPSF (blue) and NPVF (red) neuropeptides. Stars indicate sequence identity across the alignment. (**B**) Schematic of larval zebrafish, with location of NPVF neurons (*hyp*.: hypothalamus; *vRN*: ventral raphe nucleus). (**C**) Confocal Z-projection of *Tg(npvf:EGFP*) larval fish at 5 dpf. Hypothalamus and vental raphe nucleus are highlighted in Box 1 and 2 respectively. (**D**) Confocal overlay of EGFP+ neurons and NPVF peptide in the hypothalamus at 5 dpf. (**E**) eGFP+ axons and 5HT antibody stain in the raphe nucleus at 5 dpf. (**F**) Neurons in the vRN are positive for *in situ* hybridization for *gpr147* at 5 dpf. Scale bars: C = 100 μm, D, E and F = 10 μm.

NPSF and NPVF neuropeptides from the NPVF precursor share a strong sequence conservation between human, mouse and zebrafish (Fig. 1A), suggesting that their functions are conserved. To visualize these neurons, we used a compact *npvf* promoter to generate a *npvf:egfp* transgenic line that recapitulates endogenous *npvf* expression (Fig. 1B–D and Fig. sup 1B,C). Like in mammals, NPVF+ and HCRT+ cell bodies are intermingled, without overlap (Fig. sup 1; ref. 8), in the zebrafish lateral hypothalamus^9^. Despite this anatomical proximity, HCRT+ and NPVF+ cells projected to different downstream brain regions. Most notable was a prominent projection from NPVF+ cells to the ventral-posterior part of the raphe nucleus (vRN; Fig. 1E). We also found that the RFamide receptor, *gpr147*, mirrors the spatial pattern of axonal innervation in the vRN (Fig. 1F). The expression of this receptor has also been reported in the raphe nucleus of rodents^10^,^11^, suggesting a conservation of its function in vertebrates. These observations suggest a functional interaction between hypothalamic NPVF neurons and the serotonergic system.

**Figure 2:**
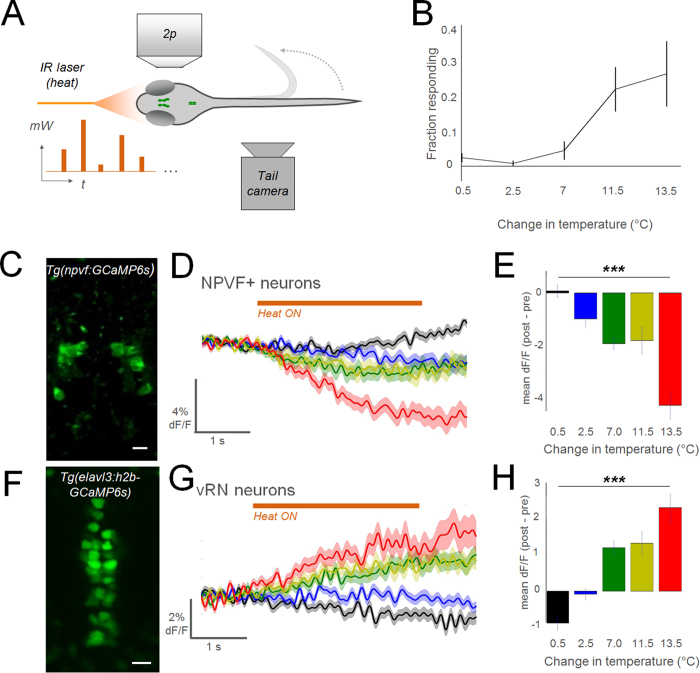
Noxious stimuli inhibit NPVF neurons and activate the vRN. (**A**) Schematic of experimental configuration (*mW*: milliwatt, output of laser; *t*: time). (**B**) Results of behavior in wildtype larvae (n = 12; mean ± SEM). (**C**) 2p imaging field of view in *Tg(npvf:GCaMP6s*) larva: average of time-series. (**D**,**G**) Summed responses of all recorded neurons in each group. Colors indicate level of heat, as denoted in summary graphs (**E**,**H**) to right (±SEM). In a single larva, multiple cells were analyzed (10 trials in each heat level, 50 trials total per larva). NPVF: n = 113 neurons in 13 larvae. vRN neurons: 173 cells in 6 larvae. (**E**,**H**) Summary data for each cell type (±SEM). 1-way anova test. ***p < 0.001. (**F**) 2p imaging field of view in *Tg(elavl3:h2b*-*GCaMP6s*) larva: average of time-series. Scale bar: 10 μm.

The NPVF systems and the vRN have both been implicated in nociception. Therefore, we next characterized the activity of NPVF and vRN cells in response to noxious stimuli. We chose a thermal nociceptive stimulus, evoked with an infrared laser to focally apply different levels of heat to head-fixed larvae^12^. We found the probability of escape-like movements increased as a function of heat (Fig. 2A,B), indicating this stimulus has an aversive effect. To record neural activity during these stimuli, we used 2-photon calcium imaging of NPVF neurons in *Tg(npvf:GCaMP6s*) larval zebrafish (Fig. 2C). We found that NPVF neurons were suppressed by thermal stimuli, with greater suppression to increasing levels of heat (Fig. 2D,E). NPVF neurons were also suppressed in response to a noxious chemical stimulus (Fig. sup 2). We next recorded from downstream vRN neurons in *Tg(elavl3:h2b*-*GCaMP6s*) zebrafish (Fig. 2F), and found that thermal stimuli increased the activity of these cells at high levels of heat (Fig. 2G,H). These activity patterns were also present in trials without tail movement (Fig. sup 3), indicating that this response is independent of locomotor activity.

**Figure 3:**
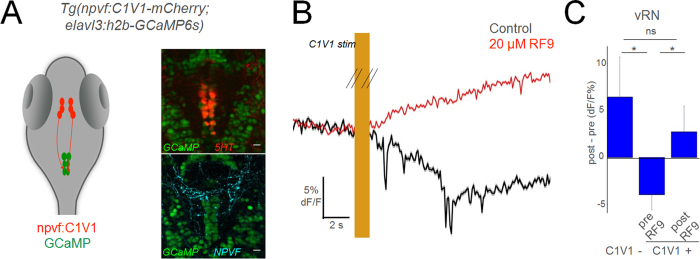
NPVF neurons in the hypothalamus inhibit serotonergic vRN. (**A**) Schematic of optogenetic experiments, and overlay of 5HT or NPVF antibody stains on GCaMP+ vRN neurons. (**B**) Mean response of vRN neurons to optogenetic activation of NPVF neurons, before and after application of gpr147 antagonist RF9. Responses are the mean of all neurons in one example larva (mean ± standard error of the mean (SEM)). (**C**) Grouped data from C1V1− (5 larvae), and C1V1+ (5 larvae) before and after RF9 application (mean ± SEM). Comparisons are 2-tailed t-tests. *p < 0.05; ns: not significant (p > 0.05).

During painful stimuli, vRN neurons are excited while NPVF neurons are suppressed. In light of the NPVF-to-vRN anatomical projection we have established, this functional data suggests that NPVF cells inhibit the vRN, and suppression of this inhibition leads to net excitation of vRN cells. We tested this hypothesis directly by combining 2-photon calcium imaging and optogenetics. We recorded activity from vRN neurons while activating NPVF neurons with the red shifted channelrhodopsin C1V1^13^, using double transgenic *Tg(npvf:C1V1*-*mCherry*; *elavl3:h2b*-*GCaMP6s*) zebrafish (Fig. 3A and Fig. sup 4A). Optical activation of NPVF neurons (Fig. sup 4B) induced a slow suppression of vRN neurons (Fig. 3B,C). This inhibition was strongly reduced in the presence of the RFamide receptor/gpr147 antagonist (RF9; ref. 14, (Fig. 3B,C and Fig. sup 4C,D), indicating that this effect is, at least partially, mediated via RFamide receptor activity.

Together, our data reveal a novel hypothalamo-raphe neural circuit interaction associated with nociception in vertebrates. This study indicates that thermal stimuli activate raphe neurons and this activation is correlated to the removal of the inhibition from hypothalamic NPVF neurons. In mammals, activation of the raphe nuclei are known to have an anti-nociceptive effect^15^,^16^, and some ventral raphe nuclei, including the ventrally located nucleus raphe magnus, project directly to the dorsal horn of the spinal cord to modulate pain^17^. Furthermore, systemic administration of RFamide receptor agonists has been reported to have a pro-nociceptive effect in rats^2^,^3^,^18^. As pain in humans is often treated by opioids, whose addictive properties have a profound negative societal impact^19^, the NPVF-serotonin circuit may be a novel target for development of alternative therapeutics for treatment of acute or chronic pain.

## Methods

### Fish lines and developmental conditions

Embryos were raised and staged according to standard protocols^20^. Our Procedures are detailed in both our “Standard Operating Procedures” and our “Protocol For Care And Use Of Laboratory Animals” (protocol #9935) reviewed and approved by the the American Association for the Accreditation of Laboratory Animal Care (AAALAC), in accordance with Stanford University animal care guidelines. The previously described line *Tg(hcrt:eGFP*)^21^ was used to visualize hypocretin neurons. *Tg(elavl3:h2b*-*GCaMP6s*) was used to visualize neural activity during live imaging of vRN^22^.

### Generation of transgenic zebrafish

For the generation of *Tg(npvf:eGFP*), *Tg(npvf:C1V1*-*mCherry*), *Tg(npvf:GCaMP6s*-*p2A*-*TagRFP*), referred as *Tg(npvf:GCaMP6s*), 2 kb of the zebrafish *npvf* promoter amplified by PCR on genomic DNA was cloned in the p5E 5′ entry vector of the tol2kit. ORFs for *C1V1*^13^ and *GCaMP6s*^23^ were cloned in the pME entry vector. The appropriate entry and middle entry clones were mixed with the SV40pA 3′ entry vector, and recombined into the Tol2 transposon destination vector^24^. To establish stable transgenic lines, plasmids were injected into one-cell stage embryos with the Tol2 mRNA transposase.

### *In situ* hybridization and immunohistochemistry

Larval fish were fixed overnight at 4 °C in 4% paraformaldehyde/1xPBS, after which they were dehydrated through an ethanol series and stored at −20 °C until use.

*In situ* hybridizations were performed as previously described^25^. *hcrt, npvf* and *gpr147* ORFs was cloned in a pCS2+ vector using zebrafish cDNA and antisense DIG labelled probes were transcribed using the linearized pCS2+ plasmid containing the ORF. *In situs* were visualized with Fast Red (Roche) as substrates.

Immunohistochemical stainings were performed as previously described^26^, using either anti-GFP (1/1000, Torrey Pines Biolabs), anti-mCherry (1/200, Abcam), anti-5HT (1/1000–1/200, ImmunoStar) and anti-NPVF (1/200, Abcam) as primary antibodies and Alexa 488, Alexa 555, Alexa 594, or Alexa 657-conjugated goat anti-rabbit IgG, goat anti-mouse IgG, or donkey anti-rabbit IgG (1/1000–1/200) as secondary antibodies (Molecular Probes).

### Imaging of fixed samples

Fixed samples were imaged using an Olympus Fluoview FVMPE-RS multiphoton microscope or a Leica SP5 confocal microscope (Stanford cell science imaging facility). Images were visualized using Fiji/ImageJ or Photoshop (Adobe) software.

### Thermal stimuli and behavioral response

For behavioral and imaging experiments, all larvae were 8 days post-fertilization. Larvae were embedded in the lid of a 35 mm petri dish, using a thin layer of 2.5% low-melting point agarose (Invitrogen) in fish system water, 1–4 hours before behavioral task. Once the agarose had settled, we carefully removed the agarose around the tail with a scalpel, to allow for tail movements. Immediately before initiation of the task, we confirmed motor activity in the larvae by observing tail response to tapping of the petri dish.

Tail movements were recorded using an AVT Manta G031B camera (Allied Vision) running at 120 Hz. To assess nociception, we used a thermal pain assay. A 980 nm laser (980M500, Dragon Lasers) was coupled to an OM1 62.5 μm core fiber (0.28 NA). The end was stripped and the raw fiber tip was placed ~0.5 mm from the dorsal-anterior end of the larva head. Laser output and behavioral recording was monitored using custom software written in Python. We measured the temperature of each laser output by embedding the end of a thermocouple device (Omega Engineering) in agarose in a petri dish, as we did with larvae. We measured temperature as a change in **°**C. Temperature was not driven to the point of extreme noxious heat, where damage to tissue occurs^27^.

### 2-photon calcium imaging

We imaged neural activity in head-fixed behaving zebrafish using 2-photon microscopy on an Olympus Fluoview FVMPE-RS multiphoton microscope. All recordings were performed using resonant scanning in a single z-plane, with frame averaging, at 9–21 Hz. We used a 25x/1.05 NA objective (Olympus). Imaged larvae were treated with PTU (0.2 mM) to prevent pigment formation and facilitate optical access.

### Optogenetics

Experiments combining imaging and optogenetics were performed using the Olympus Fluoview FVMPE-RS multiphoton microscope. We used full-field illumination through the imaging objective with a 588 nm laser at 80% output for ~3 s, in order to activate C1V1 while limiting cross-talk with GCaMP emission. Larvae were fully embedded in 2.5% agarose and z-planes with GCaMP+ cells of interest were imaged at 20 Hz. Fish were imaged for 10 repetitions with 20 s inter trial interval.

### Pharmacology

RF9 (Sigma) was dissolved in fish system water, and was used at the final concentration of 20 μM^14^. After larvae were fully embedded, the agarose around the tail was removed, and fish water was removed and replaced with 20 μM RF9 in fish water, or fish water alone (vehicle control). Imaging began 2–3 hours later. Mustard oil (allyl isothiocyanate, Sigma) was diluted in fish water to a final concentration of 1 mM^28^, and back-filled into a pipette. This solution was then pressure-ejected (10 psi, 50–100 ms) onto the exposed tail of head-embedded zebrafish during calcium imaging of NPVF neurons, every 15–20 s, for a total of 10 presentations per larva.

### Data Analysis

All analysis was performed using custom code written in Python. Raw tail movies were processed to obtain the shape and orientation of the tail for each frame. Each movement was then classified according to the pixel displacement, velocity, and maximum angle of the tail. Escape movements were separated from forward swims by using a threshold of velocity and angle. For thermal stimuli, trials were classified as evoking an escape if the escape start time occurred within a time frame of 0.5–3.5 s after stimulus onset, but no escapes occurred within the 1 s prior to stimulus onset (to exclude trials were larvae were in a state of high motor activity, rather than responding to stimuli).

To analyze neural activity, imaging planes were first motion-corrected using the TurboReg plugin in Fiji/ImageJ^29^,^30^. ROIs around cell bodies were defined manually and raw fluorescence signals were extracted. Signals from each neuron were converted into *dF/F*, using baseline *F* as the 5^th^ percentile of the entire time series. Stimulus-triggered averages were computed for each heat level or laser stimulation, using the difference in post-heat response (mean over 4 s after heat-onset) minus pre-heat response (mean over 1 s before heat-onset). In the case of optogenetics and imaging, light emission at 588 nm caused a slight global increase in PMT recordings. As vRN responses to NPVF activation was slow, we blanked the traces for the 3 s of 588 nm stimulation, and calculated the response values as post-laser responses minus pre-laser responses.

## Additional Information

**How to cite this article**: Madelaine, R. *et al*. The hypothalamic NPVF circuit modulates ventral raphe activity during nociception. *Sci. Rep.* **7**, 41528; doi: 10.1038/srep41528 (2017).

**Publisher's note:** Springer Nature remains neutral with regard to jurisdictional claims in published maps and institutional affiliations.

## Supplementary Material

Supplementary Figures and Legends
