# Supplementary material for: The hypothalamic NPVF circuit modulates ventral raphe activity during nociception
Source: Sci Rep. 2017 Jan 31;7:41528. doi: 10.1038/srep41528 (PMC5282529; doi:10.1038/srep41528)
Supplement: Supplementary Figures and Legends [file srep41528-s1.pdf]

The hypothalamic NPVF circuit modulates ventral raphe activity during nociception

Romain Madelaine[1,*], Matthew Lovett-Barron[2,*], Caroline Halluin[1], Aaron Andalman[2], Jin Liang[1], Gemini M Skariah[1], Louis C Leung[1], Vanessa M Burns[2,3], Philippe Mourrain[1,4,#]

[1]Department of Psychiatry and Behavioral Sciences, Stanford, CA 94305, USA
[2]Department of Bioengineering and CNC program, Stanford, CA 94305, USA
[3]Department of Chemical Engineering, Stanford, CA 94305, USA
[4]INSERM U1024, Ecole Normale Supérieure Paris, 75005, France

*these authors contributed equally to this work
[#]corresponding authors: mourrain@stanford.edu

SUPPLEMENTARY FIGURE LEGENDS

Supplementary Figure 1: Co-expression of *npvf* but not *hcrt* in *Tg(npvf:eGFP)* larval zebrafish
A) Confocal Z-projection of *Tg(npvf:EGFP)* larval fish at 5 dpf.
B) Confocal Z-projection of *Tg(hcrt:EGFP)* larval fish at 5 dpf.
C) Confocal z-projection from *Tg(npvf:EGFP)* and *npvf* in situ hybridization showing colocalization.
D) Confocal z-projection from *Tg(npvf:EGFP)* and *hcrt* in situ hybridization showing that Npvf+ neurons are not expressing *hcrt*.
Scale bars: 100 µm (A, B) or 10 µm (C, D)

Supplementary Figure 2: NPVF neurons activity is inhibited by chemical noxious stimuli.
A) Schematic of experimental configuration. 1 mM mustard oil was puffed from a glass pipette onto the exposed tail of head-fixed larval zebrafish.
B) Response of NPVF neurons to puff of mustard oil. Responses are the mean of all neurons in one example larva, and each neuron is the mean of 10 trials.
C) Grouped data for responses to mustard oil. Neurons grouped from 9 larvae. All neurons are averages of 10 trials.

Supplementary Figure 3: Pattern of neural responses to thermal stimuli is preserved in trials without movement.
Summary data from larvae in Fig. 2, but trials with movement during the 3s of heat stimulus are excluded. NPVF: n=113 neurons in 13 larvae. vRN neurons: 173 cells in 6 larvae.
1-way anova test. *** $p<0.001$
A) NPVF+ neurons, B) vRN neurons.

Supplementary Figure 4: Optogenetic activation of NPVF neurons using C1V1.
A) Schematic of optogenetic experiments, and overlay of mCherry and NPVF antibody stains on GCaMP+ hypothalamus neurons in *Tg(elavl3:h2b-GCaMP6s)* larvae.
B) Grouped data from C1V1- (3 larvae), and C1V1+ (10 larvae).
C) Schematic of optogenetic experiments, and mean response of vRN neurons to optogenetic activation of NPVF+ neurons, before and after application of vehicle (swapped out fish water). Responses are the mean of all neurons in one example larva.
D) Grouped data from C1V1+ fish before and after vehicle application (5 larvae for each condition).
Comparison is a 2-tailed t-test. *** $p<0.001$; ns: not significant ($p>0.05$)
Scale bars: 10 µm

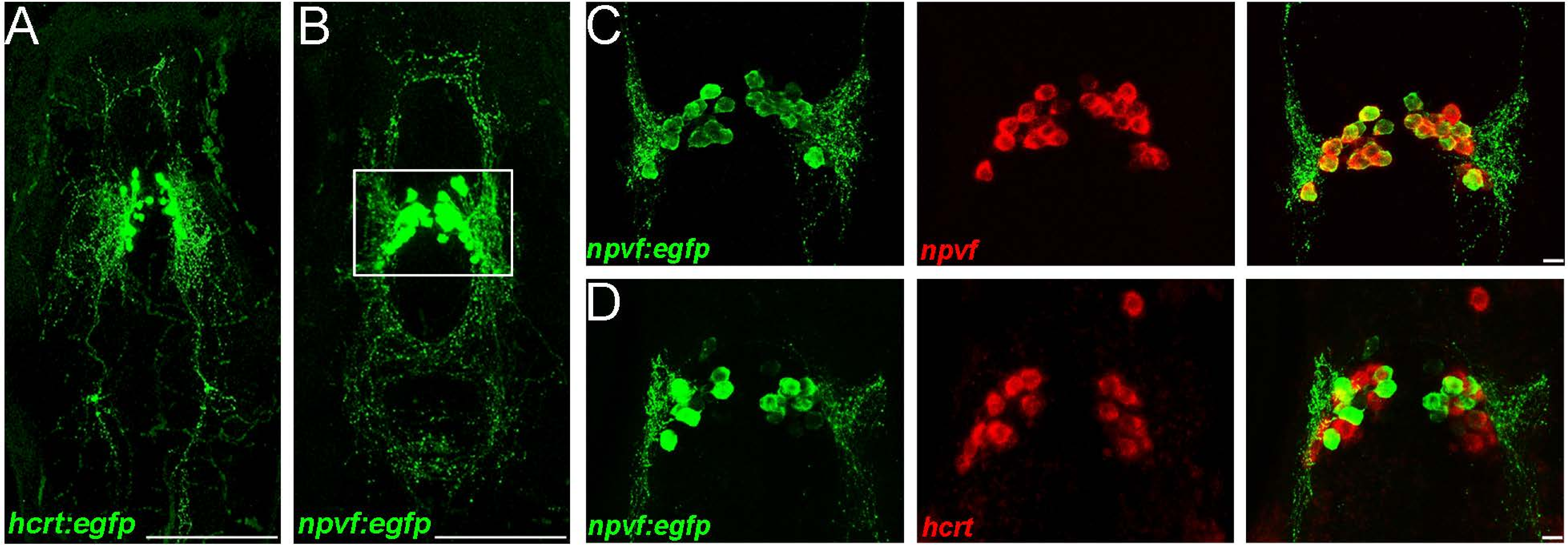

Figure S1

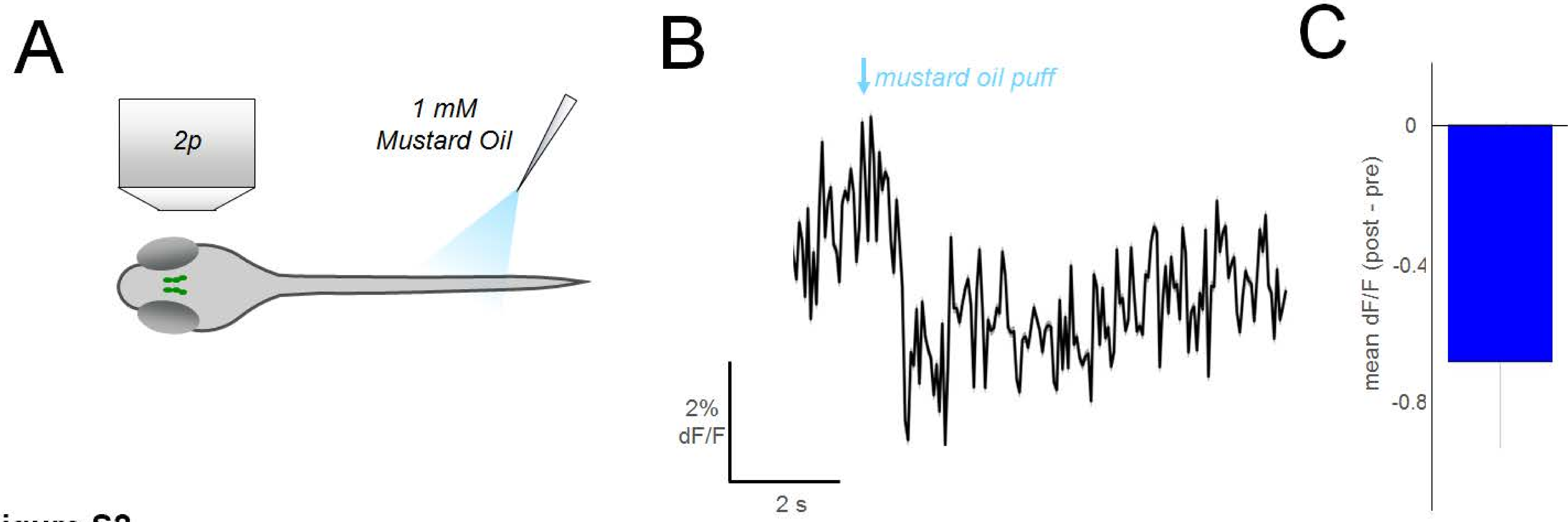

A
2p
1 mM
Mustard Oil
B
mustard oil puff
2%
dF/F
2 s
C
0
-0.4
-0.8
mean dF/F (post - pre)

Figure S2

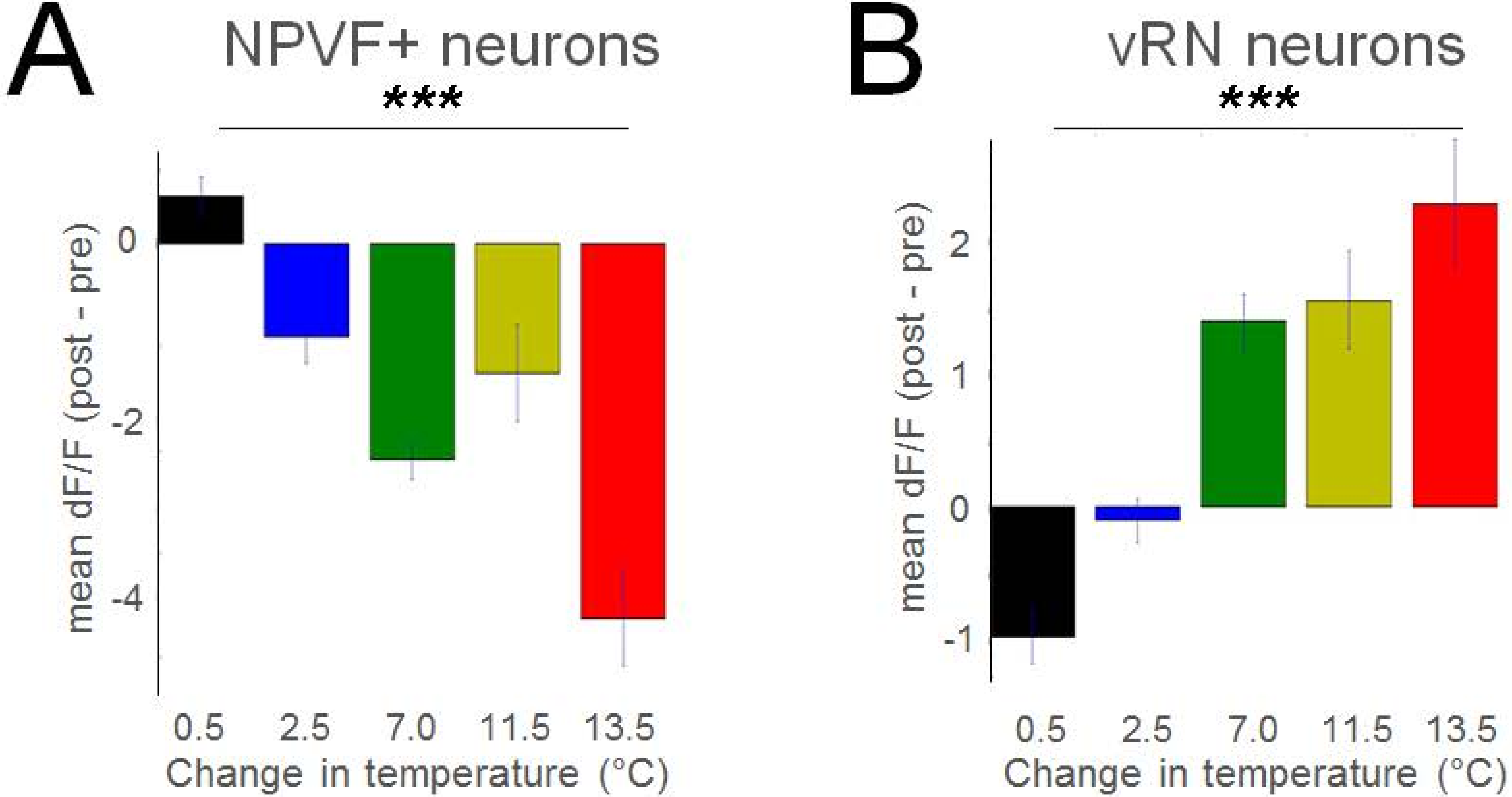
A
NPVF+ neurons
***
mean dF/F (post - pre)
0
-2
-4
0.5
2.5
7.0
11.5
13.5
Change in temperature (°C)
B
vRN neurons
***
mean dF/F (post - pre)
2
1
0
-1
0.5
2.5
7.0
11.5
13.5
Change in temperature (°C)

Figure S3

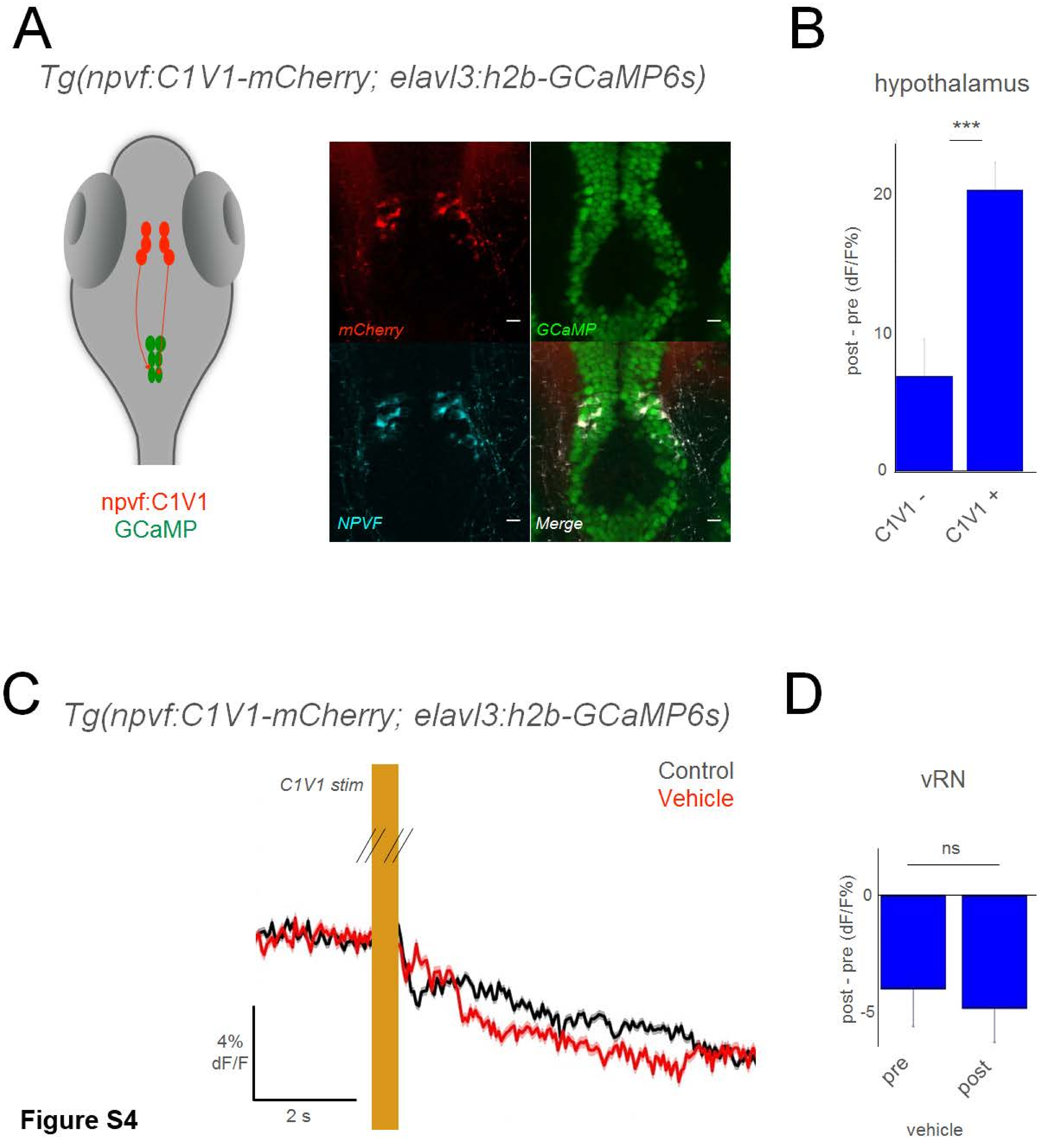
A
Tg(npvf:C1V1-mCherry; elavl3:h2b-GCaMP6s)
npvf:C1V1
GCaMP
mCherry
GCaMP
NPVF
Merge
B
hypothalamus
***
post - pre (dF/F%)
20
10
0
C1V1 -
C1V1 +
C
Tg(npvf:C1V1-mCherry; elavl3:h2b-GCaMP6s)
C1V1 stim
Control
Vehicle
4%
dF/F
2 s
D
vRN
ns
post - pre (dF/F%)
0
-5
pre
post
vehicle

Figure S4
